# Relationship between self-efficacy, self-care behaviour and glycaemic control among patients with type 2 diabetes mellitus in the Malaysian primary care setting

**DOI:** 10.1186/s12875-018-0725-6

**Published:** 2018-03-09

**Authors:** Zahirah Tharek, Anis Safura Ramli, David Leonard Whitford, Zaliha Ismail, Maryam Mohd Zulkifli, Siti Khuzaimah Ahmad Sharoni, Asrul Akmal Shafie, Thevaraajan Jayaraman

**Affiliations:** 1 Discipline of Primary Care Medicine, Faculty of Medicine, Universiti Teknologi MARA (UiTM), Selayang Campus, Jalan Prima Selayang 7, 68100 Batu Caves, Selangor Malaysia; 2 Institute of Pathology, Laboratory and Forensic Medicine (I-PPerForM), Universiti Teknologi MARA (UiTM), Sungai Buloh Campus, Jalan Hospital, 47000 Sungai Buloh, Selangor Malaysia; 3 Department of General Practice, Royal College of Surgeons in Ireland (RCSI), Mercer Building, Noel Purcell Walk, Dublin, Republic of Ireland; 4 Discipline of Population Health and Preventive Medicine, Faculty of Medicine, Universiti Teknologi MARA (UiTM), Sungai Buloh Campus, Jalan Hospital, 47000 Sungai Buloh, Selangor Malaysia; 5 Department of Family Medicine, School of Medical Sciences, Universiti Sains Malaysia (USM), 16150 Kubang Kerian, Kelantan Malaysia; 6 Department of Nursing, Faculty of Health Sciences, Universiti Teknologi MARA (UiTM), Puncak Alam Campus, 42300 Bandar Puncak Alam, Selangor Malaysia; 7 Discipline of Social and Administrative Pharmacy, School of Pharmaceutical Sciences, Universiti Sains Malaysia (USM), 11800 Penang, Malaysia; 8 Discipline of Medicine, Faculty of Medicine, Universiti Teknologi MARA, Sungai Buloh Campus, Jalan Hospital, 47000 Sungai Buloh, Selangor Malaysia

**Keywords:** Type 2 diabetes mellitus, Self-efficacy, Self-care behaviour, Primary care, Malaysia

## Abstract

**Background:**

Self-efficacy has been shown to be positively correlated with self-care behaviour and glycaemic control among patients with type 2 diabetes mellitus. However, such evidence is lacking in the Malaysian primary care setting. The objectives of this study were to i) determine the levels of self-efficacy, self-care behaviour and glycaemic control among patients with type 2 diabetes mellitus in the Malaysian primary care setting ii) determine the relationship between self-efficacy, self-care behaviour and glycaemic control iii) determine the factors associated with glycaemic control.

**Methods:**

This was a cross-sectional study involving patients with type 2 diabetes mellitus from two public primary care clinics in Malaysia. Self-efficacy and self-care behaviour levels were measured using previously translated and validated DMSES and SDSCA questionnaires in Malay versions, respectively. Glycaemic control was measured using HbA_1c._

**Results:**

A total of 340 patients with type 2 diabetes mellitus were recruited. The total mean (±SD) of self-efficacy and self-care behaviour scores were 7.33 (±2.25) and 3.76 (±1.87), respectively. A positive relationship was found between self-efficacy and self-care behaviour (*r* 0.538, *P* < 0.001). Higher self-efficacy score was shown to be correlated with lower HbA_1c_ (*r* − 0.41, *P* < 0.001). Multiple linear regression analysis demonstrated that higher self-efficacy scores (*b* − 0.398; 95% CI: -0.024, − 0.014; *P* < 0.001), shorter duration of diabetes (*b* 0.177; 95% CI: 0.002, 0.007; *P* < 0.001) and smaller waist circumference (*b* 0.135; 95% CI: 0.006, 0.035; *P* = 0.006), were significantly associated with good glycaemic control.

**Conclusion:**

This study demonstrated that higher self-efficacy was correlated with improved self-care behaviour and better glycaemic control. Findings of this study suggest the importance of including routine use of self-efficacy measures in the management of type 2 diabetes mellitus in primary care.

**Electronic supplementary material:**

The online version of this article (10.1186/s12875-018-0725-6) contains supplementary material, which is available to authorized users.

## Background

Type 2 Diabetes Mellitus (T2DM) has become a major burden not just to individuals but also to the health care systems both nationally and internationally. Globally, 387 million people have T2DM in 2014 and this is expected to rise to 592 million by 2035 [1]. The prevalence of T2DM in Malaysia has dramatically escalated over the past five decades. The Malaysian National Health Morbidity Survey (NHMS) 2011 showed an almost 20-fold increase in the prevalence of registered T2DM from 0.65% in 1960 to 15.2% in 2011 [2]. Analysis of the National Diabetes Registry (NDR) has shown that majority of patients with T2DM have co-existing cardiovascular (CV) risk factors such as hypertension which was found in 70.1% of patients followed by dyslipidaemia in 55.1% [3]. Local evidence also showed that majority (52.6%) of patients with T2DM received sub-optimal management of these CV risk factors, resulting in poor control [4]. T2DM poses a significant healthcare burden accounting for 16% of the Malaysian healthcare budget (2.4 billion Malaysian Ringgits), with the majority of resources being used to treat diabetes complications, which often arise from poor glycaemic control as well as poor control of the associated CV risk factors [5].

In Malaysia, majority of patients with T2DM are being managed in the primary care setting [6, 7]. In spite of the availability of national policies and programmes, including national clinical practice guidelines detailing treatment recommendations to improve diabetes care, the number of patients with good glycaemic control remains low [2–4].

The NDR project showed that the mean HbA_1c_ among patients with T2DM was 8.1% and only 23.8% achieved the glycaemic target of < 6.5% [3]. This highlights that it is important to look at patient factors as a mean to improve glycaemic control and the control of other associated CV risk factors to reduce complications alongside other aspects of the health care system.

Patient self-efficacy has been shown to be positively correlated with improving diabetes control via diabetes self-care [8–11]. Self-efficacy has been defined as one’s ability to perform goal-directed behaviours in the presence of an obstacle or barrier [12]. Putting this concept into the context of T2DM, the goal-directed behaviours refers to self-care behaviours including adhering to self-monitoring of blood glucose (SMBG), dietary control, physical activity, foot care and medication intake as recommended. The self-efficacy concept translates into the level of self-confidence that the patients have in performing these recommended self-care behaviours. The integration of self-efficacy theory into the Health Belief Model (HBM) proposes that self-efficacy improves self-care behaviour which ultimately leads to better glycaemic control [13, 14].

Evidence is accumulating to show that glycaemic control could be improved with better self-efficacy and self-care behaviour among patients with T2DM [8–11]. In Malaysia, the only study supporting this was conducted in a university hospital setting [15]. However, characteristics of T2DM patients and healthcare delivery system in primary care may differ from secondary care in terms of patient’s education level, health literacy and availability of trained healthcare personnel to deliver self-care support. In view of the lack of evidence in our primary care setting, there was a need to determine the role of self-efficacy and its relationship with self-care behaviour and glycaemic control among patients with T2DM in primary care. Therefore, the objectives of this study were to i) determine the levels of self-efficacy, self-care behaviour and glycaemic control among patients with T2DM in the Malaysian primary care setting ii) determine the relationship between self-efficacy, self-care behaviour and glycaemic control, and iii) determine the factors associated with glycaemic control.

## Methods

### Study design and setting

This was a cross-sectional study carried out at two public primary care clinics in the state of Selangor, Malaysia from August 2014 to September 2015. These clinics were selected because they were public primary care clinics located in urban areas with heavy patient load and good multiracial diversity. In addition, both clinics have a Non-Communicable Disease (NCD) clinic dedicated for T2DM providing a good pool for patient recruitment.

### Study population

The study population was patients with T2DM who were receiving care at the two public primary care clinics. The inclusion criteria included T2DM patients aged ≥18 years old who have been diagnosed with T2DM for at least 1 year duration, received follow-up care at the primary care clinic at least twice within the last 1 year (to establish that the patient was a regular patient at the clinic) and were able to speak and understand Malay language. The exclusion criteria included those with Type 1 Diabetes Mellitus, pregnant, mental disorders associated with a loss of a sense of reality (schizophrenia, bipolar disorder, Alzheimer’s disease, psychosis or dementia), any hearing or visual impairment that may impede patients from understanding instruction and completing the self-administered questionnaires, any literacy problems that may impede ability to give informed consent and/or any major complications that could interfere with self-care behaviours (such as being blind, suffered from debilitating strokes or coronary disease).

### Sampling method

Patients with T2DM who attended the NCD clinics at the two primary care clinics were approached consecutively by the investigators and were given a patient information leaflet describing the study and its objectives. Those who were interested to participate were screened using the eligibility checklist according to the inclusion and exclusion criteria. Written informed consent was obtained from patients who fulfilled the inclusion and exclusion criteria. Participants were informed that they were free to withdraw from the study at any time.

### Study tools

The tools for this study consisted of two sets of questionnaires which have been previously translated and validated: the Diabetes Management Self-Efficacy Scale (DMSES) Malay version [15] and the Summary of Diabetes Self-Care Activities Scale (SDSCA) Malay version [16].

The DMSES Malay version [15] was chosen to measure the self-efficacy levels in this study population because of its reliability (Cronbach’s α of 0.951) and comprehensiveness in covering the domains in diabetes management. The self-administered questionnaire consisted of 20 items with 11-point (0 to 10) Likert scale that measures 5 domains of participants’ perceived self-efficacy in diabetes self-care activities i.e. dietary control, blood glucose monitoring, physical activity, foot-care and medication intake [15]. Overall, the scores range from 0 to 200 with higher scores indicating greater levels of self-efficacy [15].

The SDSCA Malay version [16] was chosen because of its reliability (Cronbach’s α of 0.740), brevity and ease of scoring. It is a self-administered questionnaire comprising of 10 items with five subscale domains which measure the frequency of various self-care activities perform by a patient over the previous 7 days [16]. The five scale domains include general diet, specific diet, physical activity, blood glucose testing and foot-care. Response choices range from 0 to 7 giving a total score ranging from 0 to 70 [16]. Higher total scores indicate better performance of self-care behaviour [16].

The HbA_1c_ level taken within the 3 months prior to the extraction of the clinical data was used as a measure of glycaemic control of the study population. There was no HbA_1c_ missing data because patients without HbA_1c_ within the last 3 months were excluded from the study. Good glycaemic control is represented by HbA_1c_ < 6.5% in accordance with the Malaysian T2DM clinical practice guideline [17].

### Data collection and study procedures

Data was collected by the investigators and staff nurses at the NCD clinic. All investigators and the staff nurses involved in this study were trained with regards to the study procedures prior to the conduct of the study to minimize variability in the method of data collection.

### Demographic and anthropometric data collection

A standardised case report (CRF) form was used to collect socio-demographic information on the study subjects i.e. age, gender, ethnic group, educational attainment, occupation, duration of T2DM and smoking status, including the number of cigarettes smoked per day for current smokers. Those who never smoked or had quit smoking for more than 6 months were considered as non-smokers.

Anthropometric measurements included height, weight, body mass index (BMI), waist circumference (WC) and blood pressure (BP). Height and weight were measured using the Seca 769 Digital Medical Scale stadiometer. Weight was measured in light clothing, without shoes on the scale with a precision of 0.1 kg. Height was measured to 0.1 cm using the stretch stature method of the stadiometer and then converted to metres. BMI was calculated using the standard formula (weight in kg)/ (height in metres)^2^. WC was measured to the nearest 0.1 cm using non-stretchable measuring tape with the subjects standing in a relaxed position and arms at the side. The measurement was taken at the midpoint between the lower rib margin (12th rib) and the iliac crest.

BP was measured twice, 2 min apart on the right arm in sitting position, using automatic digital blood pressure monitor (Omron HEM-757). Participants were made to rest for at least 5 min before the measurements were taken. Each participant was seated upright with his/her right arm supported at the heart level. The mean of the first and second systolic and diastolic measurements was reported as the BP value for individual participant.

### Questionnaires administration

Participants were given a set of DMSES-Malay and SDSCA-Malay versions. Clear written and verbal instructions were given on how to fill up the questionnaires. They were requested to circle or tick which options suited them the most. Participants were encouraged to seek clarification from the investigators at any time should any queries arise. They were also reminded to answer the questionnaires themselves rather than getting help from their accompanying family members.

**Fig. 1:**
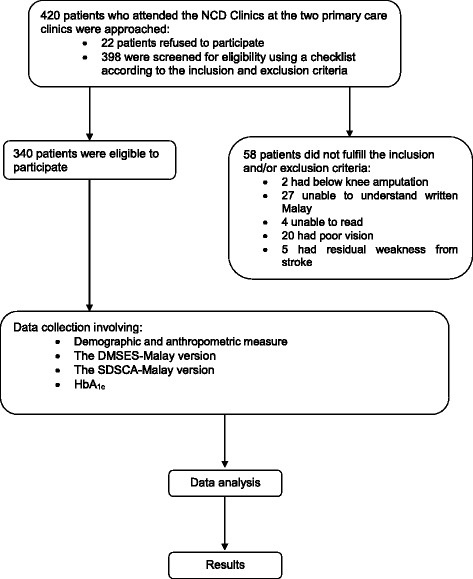
Flow chart of the conduct of the study

Participants were given a pen to complete the questionnaires at a corner of the clinic equipped with tables and chairs. The investigators ensured that participants did not interact with each other whilst answering the questionnaires. On average, participants took approximately 10 to 20 min to complete the questionnaires. Once they had finished, they handed the questionnaires to the investigators, who then checked the responses for completeness. Figure 1 illustrates the conduct of the study.

### HbA_1c_ measurement

HbA_1c_ was measured using the Bio-Rad D-10 HPLC instrument available at the study sites. HbA_1c_ level that was taken within 3 months of the data collection date was retrieved from the patients’ clinical notes.

### Sample size calculation

Sample size was calculated using the single proportion formula with 5% precision and 95% confidence interval, where the proportion (P) was estimated based on the findings of a similar study in South East Asian population which showed that 32.7% had high self-efficacy score and 32.9% had good self-care behaviour score among T2DM subjects with good glycaemic control [18]. Therefore, using Z = 1.96, ∆ (precision) = +/− 0.05, P = proportion of high self-efficacy (32.7%) or self-care behaviour (32.9%), would give a required sample size of 338 or 339, respectively. Considering additional 20% of refusal and non-eligibility rate, this study aimed to approach approximately 424 patients.

### Statistical analyses

Data was analysed using the Statistical Package for the Social Sciences (SPSS) version 21.0 (IBM). All continuous variables were described as mean (±SD) and number (n) and percentage (%) for dichotomous or nominal data. The score for item 4 of the SDSCA-Malay version was reversed as the question was negatively phrased. The levels of self-efficacy, self-care behaviour and glycaemic control were analysed using mean (±SD) as the data were normally distributed. The relationships between self-efficacy, self-care behaviour and glycaemic control were analysed using the Pearson’s correlation test since the data were normally distributed. The coefficient of correlation (*r*) ranges from + 1.0 to − 1.0, where *r* > 0 indicates positive relationship, *r* < 0 indicates negative relationship while *r* = 0 indicates no relationship [19]. A value *r* ≥ 0.8 or − 0.8 indicates strong relationship, *r* = 0.5–0.8 or − 0.5 to − 0.8 indicates moderate relationship and *r* ≤ 0.5 or − 0.5 indicates weak relationship [19]. The factors associated with glycaemic control amongst the study population were analyzed by simple linear regression followed by multiple linear regressions since the data consisted of continuous variables. The independent variables that were entered into the simple linear regression were age, gender, BMI, duration of diabetes, BP, WC, SDSCA scores and DMSES scores. Variables with a *P* value of less than 0.25 by single linear regression were then included in the multiple linear regressions and these were duration of diabetes, WC, SDSCA scores and DMSES scores. A *P* value of less than 0.05 was considered statistically significant in the multiple linear regressions.

## Results

A total of 420 patients with T2DM were approached and invited to enter the study. Out of this, 22 patients (5.2%) refused to participate and another 58 patients (13.8%) were not eligible to enter the study as they did not fulfill the inclusion and/or the exclusion criteria. Therefore, the recruitment rate for this study was 81% giving a total number of 340 eligible patients with T2DM who completed the questionnaires.

### Characteristics of the study population

Table 1 shows the sociodemographic and clinical characteristics of the participants. The majority of the participants (58.8%) were female with an average age of 58.34 years old (±11.86, range 30–89). The participants were ethnically diverse, comprising of 61.5% Malays, 19.4% Chinese, 18.2% Indians and 0.9% of other ethnicity. Almost half of the participants (47.9%) were educated up to the secondary school level. The mean HbA1c was 7.99 (±1.71). Only 13.5% had good glycaemic control (HbA_1c_ < 6.5).

**Table 1:** Sociodemographic and clinical characteristics of the patients with T2DM (*n* = 340)

Variables	n (%)	Mean (±SD)
Age (years)		58.34 (±11.86)
30–39	19 (5.6)	
40–49	63 (18.5)	
50–59	94 (27.6)	
≥ 60	164 (48.2)	
Gender		
Male	140 (41.2)	
Female	200 (58.8)	–
Ethnicity		
Malay	209 (61.5)	
Chinese	66 (19.4)	–
Indian	62 (18.2)	
Others	3 (0.9)	
Education level		
None	15 (4.4)	
Primary school (age 7 to 12)	119 (35)	–
Secondary school (age 13 to 17)	163 (47.9)	
Tertiary (college/ university)	43 (12.6)	
Occupation		
Employed	138 (40.6)	
Unemployed/Pensioner	202 (49.5)	–
Smoking status		
Smokers	40 (11.8)	–
Non-smokers	300 (88.2)	
BMI (kg/m^2^)		28.53 (±5.29)
Normal (< 23)	48 (14.1)	
Abnormal (≥ 23)	291 (85.6)	
Waist circumference (cm)		
Male		91.52 (±9.89)
Normal (< 90)	64 (45.7)	
Abnormal (≥ 90)	76 (54.3)	
Female		88.30 (±11.95)
Normal (< 80)	39 (19.5)	
Abnormal (≥ 80)	161 (80.5)	
Blood pressure (mmHg)		
Controlled (< 140/80)	135 (39.7)	–
Uncontrolled (≥140/80)	205 (60.3)	
Glycaemic control (%)		
Controlled (HbA_1c_ < 6.5)	46 (13.5)	7.99 (±1.71)
Uncontrolled (HbA_1c_ ≥ 6.5)	294 (86.5)	

### Levels of self-efficacy and self-care behaviour

Tables 2 and 3 shows the total mean scores of self-efficacy and self-care behaviour of the participants. The total mean scores (±SD) for self-efficacy and self-care behaviour were 7.33 (±2.25) and 3.76 (±1.87) respectively.

**Table 2:** The mean DMSES subscale scores among patients with T2DM (*n* = 340)

Subscale domains	Subscale items		Mean (±SD)
	Items (scale 0 = cannot do, 10 = certainly can do)		
Blood glucose monitoring	1	I am able to check my blood sugar if necessary	5.43 (±3.97)
	2	I am able to correct blood sugar when the sugar level is too high (e.g. eat different foods)	7.19 (±2.49)
	3	I am able to correct my blood sugar when the blood sugar level is too low (e.g. eat different foods)	7.19 (±2.45)
	Total mean subscale domain score		6.60 (±2.97)
Eating plan	4	I am able to choose the correct foods	7.47 (±2.09)
	5	I am able to choose different foods and stick to a healthy eating pattern	7.39 (±2.20)
	6	I am able to keep my weight under control	7.11 (±2.24)
	9	I am able to adjust my eating plan when ill	7.33 (±2.29)
	10	I am able to follow a healthy eating pattern most of the time	7.28 (±2.20)
	13	I am able to follow a healthy eating pattern when I am away from home	6.82 (±2.38)
	14	I am able to adjust my eating plan when I am away from home	7.09 (±2.20)
	15	I am able to follow a healthy eating pattern during the festive periods	6.14 (±2.57)
	16	I am able to follow a healthy eating pattern during wedding ceremonies or at a party	6.24 (±2.5)
	17	I am able to adjust my eating plan when I am feeling stressed or anxious	7.12 (±2.30)
	Total mean subscale domain score		7.00 (±1.97)
Physical activity	8	I am able to do enough exercise	7.12 (±2.3)
	11	I am able to do more exercise if the doctor advises me to	6.79 (±2.42)
	12	When I do more exercise, I am able to adjust my eating plan	6.66 (±2.44)
	Total mean subscale domain score		6.85 (±2.19)
Foot care	7	I am able to examine my feet for cuts	7.40 (±2.36)
Medications and follow-up	19	I am able to take my medication as prescribed	8.77 (±1.68)
	20	I am able to maintain my medication when I am ill	8.68 (±1.85)
	18	I am able to visit my doctor four times a year to monitor my diabetes according to treatment plan to monitor my diabetes	8.88 (±1.74)
	Total mean subscale domain score		8.78 (±1.76)
Total mean DMSES score			7.33 (±2.25)

**Table 3:** The mean SDSCA subscale scores among the patients with T2DM (*n* = 340)

Subscale domains	Subscale items		Mean (±SD)
	Self-management behaviours over the previous 7 days		
General diet	1	Follow a healthy eating plan in the last week	5.71 (±1.62)
	2	Follow a healthy eating plan (on average per week, over the past month)	5.75 (±1.53)
	Total mean subscale domain score		5.73 (±1.53)
Specific diet	3	Eats five or more servings of fruits and vegetables	5.41 (±1.83)
	4	Eats high-fat foods (reverse scoring item)	4.18 (±1.60)
	Total mean subscale domain score		4.80 (±1.18)
Physical activity	5	Participates in at least 30 min of exercise	4.60 (±2.32)
	6	Participates in specific exercise session	2.09 (±2.33)
	Total mean subscale domain score		3.35 (±1.85)
Blood glucose testing	7	Tests for blood sugar	1.18 (±1.89)
	8	Tests your blood sugar acording to the number of times recommended by your health care providers	1.19 (±1.915)
	Total mean subscale domain score		1.19 (±1.87)
Foot care	9	Check your feet	4.00 (±3.07)
	10	Inspect the inside of your shoes	3.48 (±3.18)
	Total mean subscale domain score		3.74 (±2.94)
Total mean SDSCA score			3.76 (±1.87)

Table 2 illustrates the mean scores (±SD) for each DMSES subscale domains and items. The highest subscale domain score of 8.78 (±1.76) was for ‘medications and follow-up’ and the lowest subscale score of 6.60 (±2.97) was for ‘blood glucose monitoring’.

Table 3 shows the mean subscale scores for five SDSCA subscale domains among the patients with T2DM. The highest mean subscale domain score of 5.73 (±1.53) was for ‘general diet’ and the lowest mean subscale domain score of 1.19 (±1.87) was for ‘blood glucose testing’.

### Relationships between self-efficacy with self-care behaviour and glycaemic control

Table 4 shows the correlation between self-efficacy, self-care behaviour scores and glycaemic control. There was a moderate positive correlation between self-efficacy and self-care behaviour (*r* 0.538, *P* < 0.001). A weak negative relationship was found between self-efficacy and HbA_1c_ (*r* − 0.41, *P* < 0.001). The scatter plots depicting these relationships are provided in Additional files 1 and 2.

**Table 4:** Simple linear correlation between DMSES score with SDSCA score and HbA_1c_

	DMSES score Pearson’s correlation (*r*)	*P* value^a^
SDSCA score	0.538	<0.001
HbA_1c_	−0.410	<0.001

^a^ Correlation is significant at P value < 0.01

### Factors associated with glycaemic control

Table 5 shows the multiple linear regression model summaries of factors associated with good glycaemic control. Model 3 was the best fit model as it accounted for 21.7% of the variation compared to Model 1 (16.8%) and Model 2 (19.9%). DMSES score was negatively correlated with HbA_1c_ while the duration of T2DM and WC were positively correlated.

**Table 5:** Multiple linear regression model summaries of factors associated with good glycaemic control

Variables in the model				*R* ^*2*^
	WC Coefficients (95% CI)	Duration DM Coefficients (95% CI)	DMSES Coefficients (95% CI)	
Model 1			−0.410 (−0.024, − 0.015)	0.168
Model 2		0.174 (0.002–0.007)	−0.411 (− 0.024, − 0.015)	0.199
Model 3	0.135 (0.006–0.35)	0.177 (0.002–0.007)	− 0.398 (− 0.024, − 0.014)	0.217

Table 6 presents the final multiple linear regression analysis in determining the factors associated with good glycaemic control. There were three variables with *P* < 0.05 which accounted for 21% (coefficient of determination, *R*^*2*^ = 0.210) of the variation in HbA_1c_ between individuals. These were DMSES scores, duration of T2DM and WC. The analysis demonstrated that higher self-efficacy scores (*b* − 0.398; 95% CI: -0.024, − 0.014; *P* < 0.001), shorter duration of diabetes (*b* 0.177; 95% CI: 0.002, 0.007; *P* < 0.001) and smaller waist circumference (*b* 0.135; 95% CI: 0.006, 0.035; *P* = 0.006) were significantly associated with good glycaemic control.

**Table 6:** Factors associated with good glycaemic control by multiple linear regressions

Variables	Standardized coefficients Beta (*b*) (95% CI)	t statistics	*P* value^a^
DMSES score	- 0.398 (− 0.024, − 0.014)	−8.201	< 0.001
Duration of T2DM	0.177 (0.002, 0.007)	3.664	< 0.001
Waist Circumference	0.135 (0.006, 0.035)	2.791	0.006

^a^ Multiple linear regression coefficient of determination, *R*^*2*^ = 0.210. The model was adjusted simultaneously for all variables included in the model. The model reasonably fits well. Model assumptions were met. There was no interaction and multicollinearity problem

Therefore, the final prediction equation of the model for glycaemic control among patients with T2DM was:

### Glycaemic control = 8.522–0.398*(DMSES score) + 0.177*(duration of T2DM) + 0.135*(waist circumference]

The regression analysis have shown that an increase of DMSES score by one would reduce the HbA1c by 0.398%, an increase in duration of T2DM by 1 year would increase the HbA1c by 0.177% and an increase in WC by 1 cm would increase the HbA1c by 0.135%.

## Discussion

This was the first study evaluating the relationship between self-efficacy, self-care behaviour and glycaemic control among patients with type 2 diabetes mellitus in the Malaysian primary care setting.

### Levels of self-efficacy, self-care behaviour, and glycaemic control

This study demonstrated a moderately high mean self-efficacy score (7.33) and participants were found to be most self-efficacious in tasks relating to medication intake and least self-efficacious in blood glucose testing. These findings were comparable with the study conducted in a hospital setting in Malaysia which showed a mean self-efficacy score of 7.57, where the highest score was for medication intake [15]. On the contrary, their study population was least efficacious in their eating plan [15]. Similar findings were found in a Jordanian study which showed a mean self-efficacy score of 7.26 with the highest score for efficacy to carry out medication intake [20]. Their participants were least confident in performing physical activity [20]. A likely explanation for the highest self-efficacy for medication intake is that this is a straightforward task which does not require much effort to perform. The low self-efficacy score in performing blood glucose testing in our study population highlighted the need to educate patients on self-monitoring of blood glucose (SMBG) to increase their self-efficacy to perform this task.

A moderate level of self-care behaviour (3.76) was found among the participants in our study. Participants reported practicing general dietary restriction the most and blood glucose testing the least. It is also interesting to note that blood glucose testing was also the task which the participants in this study were found to be least self-efficacious to perform. These findings were comparable to the Jordanian study which found similar level of self-care behaviour with a mean of 3.74, and blood glucose testing was also found to be the least frequently reported self-care behaviour in their study population [20]. The low self-care behaviour for blood glucose testing in our study population may be explained by the unavailability of affordable glucometer or glucose strips, even among those who are on insulin. These equipments are not available on prescriptions in public primary care clinics in Malaysia. Findings of this study further highlights the need to increase the availability of affordable blood glucose testing equipments especially for those on insulin, as well as to enhance self-behaviour skills to perform this task.

Our study highlights that only 13.5% of the participants achieved the glycaemic target of < 6.5% with mean HbA_1c_ of 7.99%, indicating difficult challenges physicians face in achieving glycaemic control among patients with T2DM in primary care. This finding is similar to the local study conducted in a teaching hospital which showed that only 15.5% of their study population achieved HbA1c of < 6.5% [15]. However, our finding is lower than the NDR report in 2012 which showed that 23.8% of patients with T2DM achieved good glycaemic control [3]. These findings highlight that our T2DM population largely has poor glycaemic control which leads to high complication rates. Rigorous efforts should focus on finding cost-effective methods to improve glycaemic control among patients with T2DM. Identification of factors associated with poor glycaemic control would help clinicians and policy makers to strategise for effective interventions.

### Relationships between self-efficacy with self-care behaviour and glycaemic control

A positive correlation between self-efficacy and self-care behaviour was demonstrated in patients with T2DM in this study, and this finding is similar to the study conducted in a hospital setting in Malaysia [15]. This is further supported by several other studies showing that high self-efficacy level was associated with better self-care behaviour [21, 22]. A review on the role of self-efficacy in diabetes care showed that self-efficacy provides a suitable framework for understanding and predicting commitment towards self-care behaviours and effectiveness of self-management in diabetes treatment [23].

A negative relationship was found between self-efficacy and HbA_1c_ in this study. This indicates that higher self-efficacy scores were significantly correlated with better glycaemic control. Similar findings were demonstrated in a study amongst Turkish patients with T2DM which showed that self-efficacy had a modest negative correlation with glycaemic control [24]. Another study proved that an increase in diabetes self-efficacy over time was related to an improvement in glycaemic control [25]. A cross-sectional study in Myanmar has shown that patients with a high self-efficacy level were 5.29 times more likely to have better glycaemic control than those with a fair or low self-efficacy level [18].

### Factors associated with good glycaemic control

This study shows that better self-efficacy, shorter duration of T2DM and smaller WC were significantly associated with good glycaemic control. Similar findings were identified by a study in Jordan where an increased duration of diabetes, not following eating plan as recommended by dietitians, negative attitude towards diabetes, and increased barriers to adherence scale scores were associated with poor diabetic control [25]. From this result, it can be expected that patients with low self-efficacy and longer duration of diabetes would have poor glycaemic control. Therefore intervention should be targeted in these groups of patients.

### Strengths and limitations of the study

The main strength of this study is the novelty of its findings in demonstrating significant relationships between self-efficacy, self-care behaviour and glycaemic control among patients with T2DM in the Malaysian primary care setting. Additional strength is the utilisation of valid and reliable tools which have been translated and validated for the Malaysian population. Limitations of this study include the non-probability sampling method which could be vulnerable to sampling bias. However, efforts were made to invite all patients with T2DM in the waiting area to participate in this study during the data collection period. This study also selected T2DM patients who received follow-up care at the clinic at least twice within the last 1 year to establish that the patients were regular patients at the clinic. This was to ensure that subsequent intervention can be targeted towards this group of patients. Self-efficacy, self-care behaviour and glycaemic control may be better in this group of patients compared to the defaulters. Findings of this study may not be generalisable to other primary care clinics in Malaysia as it was conducted in two primary care clinics in urban areas. Other limitations include the self-report method used to measure self-care behaviour, rather than more direct measures such as direct observations of the self-care behaviours. However, although self-report provides an estimate of health behaviours, it represents the most practical method of health behaviour measurement [26]. Finally, this study did not explore other potential factors which may affect glycaemic control such as education level, smoking status, presence of co-morbidities, types of medications, dietary intake and physical activity. Therefore, the multiple linear regression results should be interpreted with caution.

### Implications for clinical practice and future research

This study shows that better self-efficacy is a major determinant of good glycaemic control. Findings of this study suggest the importance to include routine use of self-efficacy measures in the management of T2DM in primary care aiming to improve glycaemic control. Assessment of self-efficacy in patients with T2DM should be an important first step in the development of individually tailored interventions. These interventions should also focus on enhancing self-efficacy to improve self-management of diabetes. Efforts should be made by primary care providers to enhance their patients’ self-efficacy in order to improve their self-care behaviour, and ultimately, glycaemic control. Primary care providers should be trained to provide self-management support to their patients with T2DM in order to increase self-efficacy and self-care practice. However, further research using systematic random sampling of patients with T2DM in a larger number of public primary care clinics in Malaysia is needed to confirm the findings of this study.

Self-efficacy and self-care behaviour of patients with T2DM would also vary over time and investigating such variations is beyond the scope of this study. Future research should include prospective cohort studies to investigate the longitudinal causal effects of self-efficacy on changes in diabetes self-care behaviour and glycaemic control. In addition, further research should also explore facilitators and barriers influencing self-efficacy and self-care behaviour. Such evidence is required to guide policy change and resource allocations in the Malaysian public primary care setting.

## Conclusions

In conclusion, this study has demonstrated that higher self-efficacy was correlated with better self-care behaviour and glycaemic control. Higher self-efficacy score, shorter duration of T2DM and smaller WC were identified as significant predictors of good glycaemic control. Despite its limitations, this study is the only study which explored such relationships among T2DM patients in the Malaysian primary care setting. Findings of this study highlight the importance to measure self-efficacy in order to develop individual self-management intervention programmes for patients with T2DM in primary care, with the aim of improving glycaemic control and reducing major complications.

## Additional files

Additional file 1: Relationship between self-efficacy and self-care behaviour scores. (DOCX 117 kb)

Additional file 2: Relationship between self-efficacy scores and HbA1c. (DOCX 103 kb)

## Abbreviations

BMI: Body mass index
BP: Blood pressure
CRF: Case report form
CV: Cardiovascular
DMSES: Diabetes Management Self-Efficacy Scale
HbA_1c_: Glycated Haemoglobin
HBM: Health Belief Model
NCD: Non-Communicable Disease
NDR: National Diabetes Registry
NHMS: National Health Morbidity Survey
SDSCA: Summary of Diabetes Self-Care Activities Scale
SMBG: Self-monitoring of blood glucose
T2DM: Type 2 Diabetes Mellitus
WC: Waist circumference
