# Supplementary figures and images for: Relationship between self-efficacy, self-care behaviour and glycaemic control among patients with type 2 diabetes mellitus in the Malaysian primary care setting
Source: BMC Fam Pract. 2018 Mar 9;19:39. doi: 10.1186/s12875-018-0725-6 (PMC5845324; doi:10.1186/s12875-018-0725-6)

**
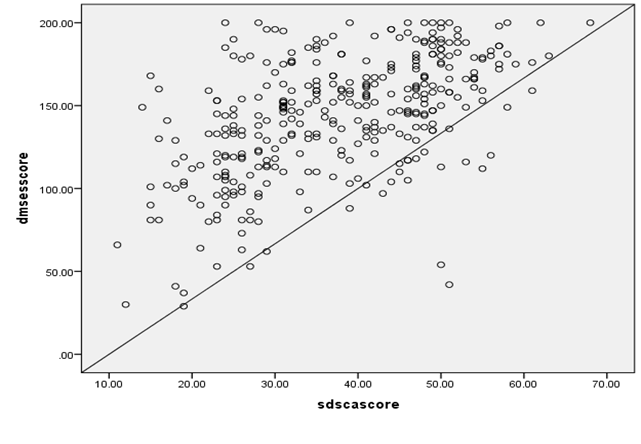
**

***r* 0.538, *P*<0.001**

Supplement: Supplementary file 1 — Relationship between self-efficacy and self-care behaviour scores. (DOCX 117 kb) [file 12875_2018_725_MOESM1_ESM.docx]

**
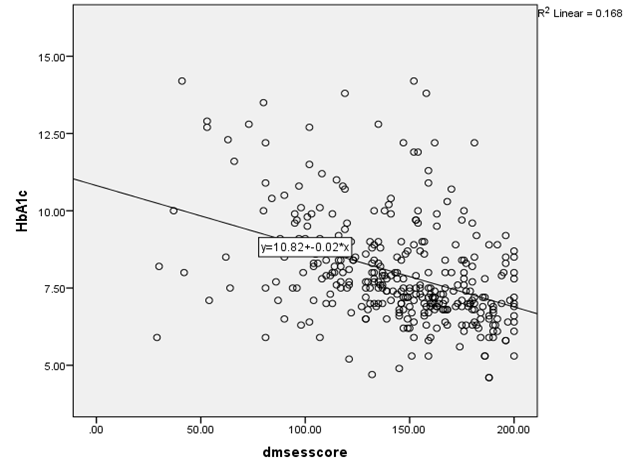
**

***r* -0.41, *P*<0.001**

Supplement: Supplementary file 2 — Relationship between self-efficacy scores and HbA1c. (DOCX 103 kb) [file 12875_2018_725_MOESM2_ESM.docx]
